# Sustained Hypothetical Interventions on Midlife Alcohol Consumption in Relation to All-Cause and Cancer Mortality: The Australian Longitudinal Study on Women’s Health

**DOI:** 10.1093/aje/kwad164

**Published:** 2023-07-24

**Authors:** Yi Yang, Allison M Hodge, Brigid M Lynch, Pierre-Antoine Dugué, Elizabeth J Williamson, Harindra Jayasekara, Gita Mishra, Dallas R English

**Keywords:** alcohol consumption, mortality, target trial emulation, women’s health

## Abstract

No randomized controlled trial has evaluated the effect of long-term alcohol interventions on mortality. Results reported in existing observational studies may be subject to selection bias and time-varying confounding. Using data from the Australian Longitudinal Study on Women’s Health 1946–1951 birth cohort, collected regularly from 1996–2016, we estimated all-cause and cancer mortality had women been assigned various alcohol interventions (in categories ranging from 0 to >30 g/day ethanol, or reduced to ≤20 g/day if higher) at baseline, and had they maintained these levels of consumption. The cumulative risks for all-cause and cancer mortality were 5.6% (10,118 women followed for 20 years) and 2.9% (18 years), respectively. For all-cause and cancer mortality, baseline ethanol up to 30 g/day showed lower risk and >30 g/day showed higher risk relative to abstention. Had women sustainedly followed the interventions, a similar relationship was observed for all-cause mortality. However, the negative association observed for intakes ≤30 g/day and positive association for intakes >30 g/day was not evident for cancer mortality. Our findings suggest that all-cause mortality could have been lower than observed if this cohort of women had consumed some alcohol (no more than 30 g/day) rather than no consumption, but cancer mortality might not.

## Abbreviations

ALSWH: Australian Longitudinal Study on Women’s Health
CI: confidence interval
HR: hazard ratio
RD: risk difference
RR: risk ratio

No randomized controlled trial has evaluated the effect of long-term alcohol interventions on mortality due to limited feasibility and ethical considerations. Evidence from observational studies consistently suggests that high alcohol consumption is associated with increased mortality (1, 2). For women aged 50 years or older, cancers accounted for an estimated 27% of total alcohol-attributable deaths in 2016, based on existing observational studies (3). Some studies showed lower all-cause mortality for light to moderate consumption (10–20 g/day of ethanol for women, 20–40 g/day for men) compared with abstention (“J-shaped” association) (4, 5), but criticisms remain that the relationship is likely due to biases (6). For example, other characteristics of light to moderate drinkers, such as higher socioeconomic status, may explain their lower mortality compared with abstainers. Furthermore, the reduced mortality in light to moderate drinkers might be partly due to the inclusion of former drinkers with abstainers in the reference category, as some former drinkers may have quit drinking alcohol because of health problems (7–9).

**Figure 1:**
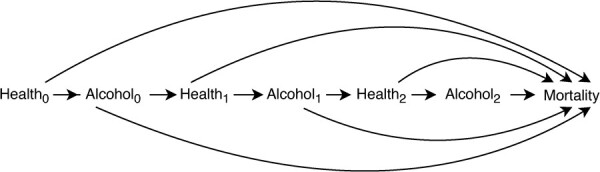
Causal diagram: General health is a determinant of both alcohol drinking and mortality. When estimating the effect of sustained alcohol exposure on risk of death in a regression model, conditioning only on baseline health status does not control for time-varying confounding from health deterioration during follow-up. However, conditioning on time-varying general health will block some of the causal pathways from alcohol to death that are mediated through postbaseline general health.

It has been suggested that alcohol consumption measured at multiple time points should be preferred over single-time-point measures because consumption changes over time. Misclassification of exposure could bias the estimate of the effect on health outcomes (10, 11). Although observational studies collecting alcohol data at multiple time points provide more information on exposure over time, the data analysis poses other challenges. In such studies, selection of participants into the analysis would be affected by both the exposure (heavy drinkers are less likely to survive to later data collection waves) and the outcome (participants who died before the last data collection are excluded). This would introduce selection bias when estimating the effect of alcohol drinking on mortality. Adjustment of time-varying confounding is also a challenge when alcohol consumption is measured repeatedly (see Figure 1) (12).

In this study, we aimed to emulate a target trial (13) to estimate the effect of sustained alcohol interventions initiated in midlife on all-cause and cancer mortality using data from a prospective study of women, while reducing selection bias and time-varying confounding.

## METHODS

### The cohort

The Australian Longitudinal Study on Women’s Health (ALSWH) is a population-based cohort study examining the health of Australian women. Three nationally representative cohorts of women (born in 1973–1978, 1946–1951, and 1921–1926) were recruited in 1996. Our study used data from the 1946–1951 birth cohort. Approximately 53%–56% of women invited completed a questionnaire about their lifestyle and health (*n* = 13,714). Further data collection occurred every 2–3 years with paper or Web-based questionnaires in 1998 (*n* = 12,338), 2001 (*n* = 11,226), 2004 (*n* = 10,905), 2007 (*n* = 10,638), 2010 (*n* = 10,011), 2013 (*n* = 9,151), and 2016 (*n* = 8,622). Details about the cohort have been reported (14, 15). We used the 1998 wave as time zero (referred to as baseline hereafter) to be able to adjust for prebaseline (1996) confounders.

Women who did not return the baseline questionnaire (*n* = 1,376) or had unknown death date (*n* = 1) were excluded. To reduce potential reverse causation from baseline ill health, we excluded participants who, at baseline, reported “fair” or “poor” general health (*n* = 1,508; as opposed to “good,” “very good,” or “excellent”), had prior cancer diagnosis or treatment (*n* = 613), or had missing information for the 2 variables (*n* = 98). These exclusions left 10,118 eligible women (see Web Figure 1, available at https://doi.org/10.1093/aje/kwad164, for flow of participants).

The study methods of ALSWH were approved by the Human Research Ethics Committees of the Universities of Newcastle and Queensland.

### Alcohol data

Alcohol data were obtained from 2 questions on usual intake: “How often do you usually drink alcoholic beverages?” and “On a day when you drink alcoholic beverages, how many drinks do you usually have?” Daily alcohol intake was approximated using frequency and quantity of consumption because type of beverage consumed was not asked. Each reported drink was assumed to be 1.5 Australian standard drinks. Each standard drink contains 10 grams of alcohol (16). For responses reporting a range of frequencies or quantities, the midpoint was used as a multiplying factor (Tables 1 and2) (17). Daily intake (grams of ethanol) was calculated as follows:

\begin{align*} Alcohol\ intake\ \left(g/ day\right)&=F\times Q\times 10\ \left(g/ standard\ drink\right)\\&\quad\div 7\ \left( days/ week\right), \end{align*}

where *F* is the weekly frequency of alcohol consumption (days/week), and *Q* is the number of standard drinks on a day of drinking. Daily alcohol intake was then categorized into 5 levels (g/day): 0, 0.1 to 10.0, 10.1 to 20.0, 20.1 to 30.0, and >30.0.

**Table 1:** Responses to the Question Used to Approximate Frequency of Alcohol Consumption^a^, Australian Longitudinal Study on Women’s Health 1946–1951 Birth Cohort, 1996–2016

**Response**	**Frequency** ^**b**^
Never or former	0
I rarely drink	0.25
Less than once	0.50
1 or 2 days	1.50
3 or 4 days	3.50
5 or 6 days	5.50
Every day	7.00

^a^ “How often do you usually drink alcoholic beverages every week?”

^b^ Days/week. For responses reporting a range, the midpoint is used as a multiplying factor.

**Table 2:** Responses of the Used to Approximate Quantity of Alcohol Consumption^a^, Australian Longitudinal Study on Women’s Health 1946–1951 Birth Cohort, 1996–2016

**Response**	**No. of Reported Drinks** ^**b**^	**No. of Standard Drinks** ^**c**^
I don’t drink	0	0
1 or 2 drinks	1.50	2.25
3 or 4 drinks	3.50	5.25
5–8 drinks	6.50	9.75
≥9 drinks	9.00	13.50

^a^ “On a day when you drink alcoholic beverages, how many drinks do you usually have?”

^b^ For responses reporting a range, the midpoint is used as a multiplying factor.

^c^ One reported drink is approximately 1.5 Australian standard drinks.

### Mortality data

Deaths were ascertained through record linkage to the Australian National Death Index. Underlying cause of death was defined according to the *International Statistical Classification of Diseases and Related Health Problems*, *10th Revision* (codes C00–C97 for cancer deaths). All-cause mortality and cause of death data were complete to December 31, 2018, and December 31, 2016, respectively.

### Target trial emulation

Table 3 describes the emulation of a target trial that aims to estimate the effect of sustained alcohol interventions on 20-year all-cause and 18-year cancer mortality among Australian women aged 47–52 years (13, 18, 19).

**Table 3:** Target Trial Emulation, Using Data From the Australian Longitudinal Study on Women’s Health 1946–1951 Birth Cohort, 1996–2016

**Protocol Component**	**Target Trial Specification**	**Emulation Using Data from the 1946–1951 Cohort**
Aim	To estimate the effect of sustained alcohol interventions on 20-year all-cause mortality and 18-year cancer mortality in Australian women aged 47–52 years	Same
Eligibility	Australian women aged 47–52 years in 1998	Same
Alcohol interventions	Participants are randomly assigned to have the following intake levels throughout the follow-up period: • 0 g/day • 0.1 to 10.0 g/day • 10.1 to 20.0 g/day • 20.1 to 30.0 g/day • >30.0 g/day • Reduce drinking to at most 20 g/day if currently drinking more than 20 g/day	Same
Assignment procedure	Randomization to one of the alcohol drinking interventions	To emulate the random assignment of interventions, we adjusted for confounders required to approximate exchangeability of the groups (Web Table 1).
Start of follow-up	Time of randomization	Return date of the 1998 questionnaire
End of follow-up	Death, or administrative end of follow-up, whichever came first	Same. Administrative end of follow-up was the date when mortality data were complete: All-cause mortality data were complete to December 31, 2018; cancer mortality data were complete to December 31, 2016.
Outcome	Deaths due to any cause within 20 years of intervention assignment; deaths due to cancer within 18 years of intervention assignment	Deaths due to any cause within 20 years after baseline; deaths due to cancer within 18 years after baseline
Causal contrast of interest	Per-protocol effect (effect that would have been observed had all women adhered to their assigned intervention over the follow-up)	Observational analogues to the intention-to-treat and the per-protocol effect
	Intention-to-treat effect (effect of being assigned to an alcohol intervention)	
Statistical analysis	Intention-to-treat analysis: Estimation can be done through comparing the outcomes of the groups assigned to each intervention.	Baseline analysis: Intention-to-treat analysis is rarely possible in observational studies. A close analogue of the intention-to-treat effect is a comparison of baseline values of the different intervention strategies, assuming adequate adjustment for baseline confounders.
	Per-protocol analysis: Estimation of the per-protocol effect requires adjustment for baseline and postbaseline prognostic factors associated with adherence to the assigned intervention. Because postbaseline prognostic factors associated with subsequent adherence to the assigned intervention may be affected by prior adherence, g-methods are required (21).	Per-protocol analysis: Adjustment for baseline and postbaseline confounding is necessary when exposure to alcohol is sustained over time. Because postbaseline confounders are affected by prior exposure, g-methods are required.

### Statistical analysis

#### Baseline alcohol and mortality.

We fitted Cox regression models with age as the timescale to estimate hazard ratios (HRs) and 95% confidence intervals (CIs) for all-cause and cancer mortality associated with baseline alcohol intake, regardless of whether the individuals continued with the same intake after baseline (referred to as baseline analysis hereafter). Follow-up began at the return date of baseline questionnaire and ended at date of death or date when mortality data were complete, whichever came first. The models included time-fixed confounders (measured before baseline, to establish a clear temporal ordering of the confounders and exposure (20): country of birth, highest educational qualification, alcohol consumption (in late teens, twenties, and thirties), having smoked at least 100 cigarettes in total prior to baseline, and self-reported history of depression or anxiety, heart disease, hypertension, and diabetes). We also included prebaseline values of the time-varying confounders (living in most socioeconomically disadvantaged areas (21), marital status, physical activity, daily number of cigarettes smoked by current smokers (zero if participants reported not smoking), body mass index, and subjective general health). See Web Table 1 for detailed units and categories.

We investigated the proportional hazards assumption by including terms for interaction between time and the covariates. In the case of evidence for a violation (*P* < 0.05), the interaction term for the corresponding variable remained in the model.

#### Observational analog to per-protocol analysis.

We used the parametric g-formula to estimate the observational analogs to the per-protocol effects of the interventions listed in Table 3 (i.e., the effect had all women sustainedly followed the assigned intervention on all-cause and cancer mortality), referred to as per-protocol analysis hereafter. The parametric g-formula is a generalization of standardization to time-varying exposures and confounders that handles time-varying confounding appropriately when the time-varying confounders are affected by prior alcohol consumption (12).

We fitted parametric models for the conditional distribution of time-varying variables from each wave. To ensure correct temporal ordering, we used confounder data collected 1 wave prior to exposure measurement to ensure they were not intermediate variables (20). All models also included time-fixed confounders, before baseline and the most recent value of the time-varying confounders. A pooled-over-interval logistic model was fitted to estimate the conditional discrete-time hazard of death for each time interval (22, 23). For cancer mortality, death due to other causes was treated as a competing event. A logistic model was fitted to estimate the conditional discrete-time hazard of death due to other causes at each time interval, which was necessary to compute the subdistribution cumulative incidence function (see Web Table 1 for details of covariates and models) (22). We assessed the robustness of our estimates to various covariate orders when modeling the joint distribution of time-varying covariates.

We then used the above models to predict the outcome had the alcohol intake been set to a value determined by the interventions using a Monte Carlo sample of 20,000 women drawn with replacement from the existing sample. We calculated risk ratios (RRs) and risk differences (RDs) using “no intervention” as the referent. Nonparametric bootstrapping (500 resamples) was used to obtain 95% CIs.

#### Imputation of missing data.

Multiple imputation using chained equations (MICE) was used to impute missing data due to incomplete questionnaire responses or nonresponses from women who were alive, assuming data were missing at random (details are presented in Web Appendix 1) (24, 25).

The parametric g-formula analysis was conducted using SAS, version 9.4 (SAS Institute, Inc., Cary, North Carolina), using the GFORMULA 3.0 macro (22). Other analyses were conducted using Stata, version 14.2 (StataCorp LP, College Station, Texas).

## RESULTS

Baseline characteristics of the 10,118 eligible women according to alcohol intake categories are presented in Table 4. The proportion of current smokers increased as the alcohol intake increased. Physical activity was lowest in the 2 extremes of intake (0 g/day and >30 g/day). Mean body mass index was lower and subjective general health was better in the “10.1–20.0 g/day” and “20.1–30.0 g/day” groups than in other alcohol-consumption categories. The abstainer and the “0.1–10.1 g/day” groups had higher percentages of women without a formal educational qualification and living in the most socioeconomically disadvantaged areas than higher intake groups. The “10.1–20.0 g/day” and “20.1–30.0 g/day” groups had proportionally fewer women in the most socioeconomically disadvantaged areas, and more women who had a diploma or higher qualification and were more physically active. The “>30.0 g/day” group had proportionally more former and current smokers, and a higher mean number of cigarettes consumed by current smokers than other groups. This group also had higher percentages of women with a history of depression or anxiety and hypertension.

**Figure 2:**
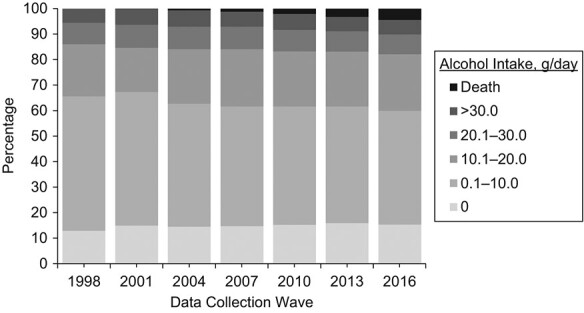
Alcohol intake over time in the 10,118 eligible women under no intervention, using data from the Australian Longitudinal Study on Women’s Health 1946–1951 Birth Cohort, 1996–2016. This figure is based on multiply imputed data set of the 10,118 women who were eligible at baseline. Percentages are averaged over 30 imputations. Numbers of all-cause and cancer deaths at each data collection wave are presented in Web Table 4.

Figure 2 gives an overview of alcohol intakes over time and the cumulative number of deaths at each wave. The percentage of abstainers increased from 13% in 1998 to 15% in 2001 and remained relatively stable thereafter. About half (53%) of the women were in the “0.1–10.0 g/day” group at baseline. This number gradually decreased over time to 45% in 2016. The percentage of women in the “10.1–20.0 g/day” group remained around 20%–22% except for the year 2001 (17%). Percentages of women in the “20.1–30.0 g/day” and “>30.0 g/day” groups were relatively stable.

**Table 4:** Baseline Characteristics of Eligible Women According to Baseline Alcohol Intake, Australian Longitudinal Study on Women’s Health 1946–1951 Birth Cohort, 1996–2016

	**Baseline Alcohol Intake (g/day)**									
	**0 (*n* = 1,176)**		**0.1–10.0 (*n* = 4,815)**		**10.1–20.0 (*n* = 1,926)**		**20.1–30.0 (*n* = 799)**		**>30.0 (*n* = 528)**	
**Characteristic** ^**a**^	**No.**	**%**	**No.**	**%**	**No.**	**%**	**No.**	**%**	**No.**	**%**
Born in Australia	875	75.4	3,640	76.2	1,496	78.3	615	77.8	413	79.0
Married or de facto	994	85.1	3,962	82.8	1,643	85.8	693	87.0	437	83.4
Highest educational qualification										
No formal qualification	261	22.4	766	16.0	188	9.8	100	12.6	73	14.0
School certificate	582	49.9	2,411	50.4	875	45.8	385	48.6	256	48.9
Diploma/university/higher degree	324	27.8	1,603	33.5	849	44.4	307	38.8	194	37.1
Area-based Index of Relative Socio-Economic Disadvantage										
Most disadvantaged quartile	308	26.2	1,179	24.5	389	20.2	163	20.4	121	22.9
Smoking status										
Never	844	72.0	2,911	60.7	1,031	53.7	338	42.4	160	30.4
Former	187	15.9	1,198	25.0	632	32.9	283	35.5	197	37.4
Current	142	12.1	684	14.3	256	13.3	176	22.1	170	32.3
No. of cigarettes per day for current smokers^b^	20 (15, 25)		20 (12, 25)		18 (10, 20)		20 (10, 25)		25 (15, 30)	
Physical activity										
Very low (<33.3 MET-minutes/week)	224	19.9	738	15.8	235	12.4	112	14.4	100	19.6
Low (33.3 to 499.9 MET-minutes/week)	318	28.3	1,389	29.8	483	25.5	192	24.6	141	27.6
Moderate (500.0 to 999.9 MET-minutes/week)	248	22.1	1,125	24.2	476	25.1	192	24.6	113	22.1
High (≥1,000 MET-minutes/week)	333	29.7	1,405	30.2	702	37	283	36.3	157	30.7
Body mass index^c^^,^^d^	27 (6.0)		27 (5.3)		25 (4.2)		25 (4.2)		26 (4.6)	
Subjective general health										
Excellent	149	12.7	645	13.4	362	18.8	141	17.6	65	12.3
Very good	445	37.8	2,044	42.5	907	47.1	376	47.1	238	45.1
Good	582	49.5	2,126	44.2	657	34.1	282	35.3	225	42.6
Ever had the following conditions										
Depression or anxiety	111	9.4	475	9.9	183	9.5	83	10.4	67	12.7
Heart diseases	10	0.9	18	0.4	12	0.6	6	0.8	2	0.4
Hypertension	103	8.8	387	8.0	141	7.3	56	7	67	12.7
Diabetes	21	1.8	40	0.8	8	0.4	5	0.6	1	0.2

Abbreviations: IQR, interquartile range; MET, metabolic equivalent; SD, standard deviation.

^a^ This table uses the data before multiple imputation.

^b^ Values are expressed as median and IQR.

^c^ Weight (kg)/height (m)^2^.

^d^ Values are expressed as mean and SD.

During an average of 20 years of follow-up, 569 women died, and during an average of 18 years of follow-up, 292 died from cancer. Proportionally more women who died reported not drinking, being physically inactive, currently smoking, and having comorbidities at baseline than those who were alive during follow-up (Web Table 2).

Table 5 shows HRs for all-cause and cancer mortality associated with baseline alcohol intake, regardless of whether women maintained the same intake afterwards. Although the HRs had wide CIs, the point estimates suggested that baseline alcohol intake up to 30 g/day was associated with reduced all-cause mortality compared with abstention, while consumption of more than 30 g/day was associated with higher all-cause mortality. Similarly, the point estimates suggested that cancer mortality was lower in women with intakes up to 30 g/day, with wide CIs.

**Figure 3:**
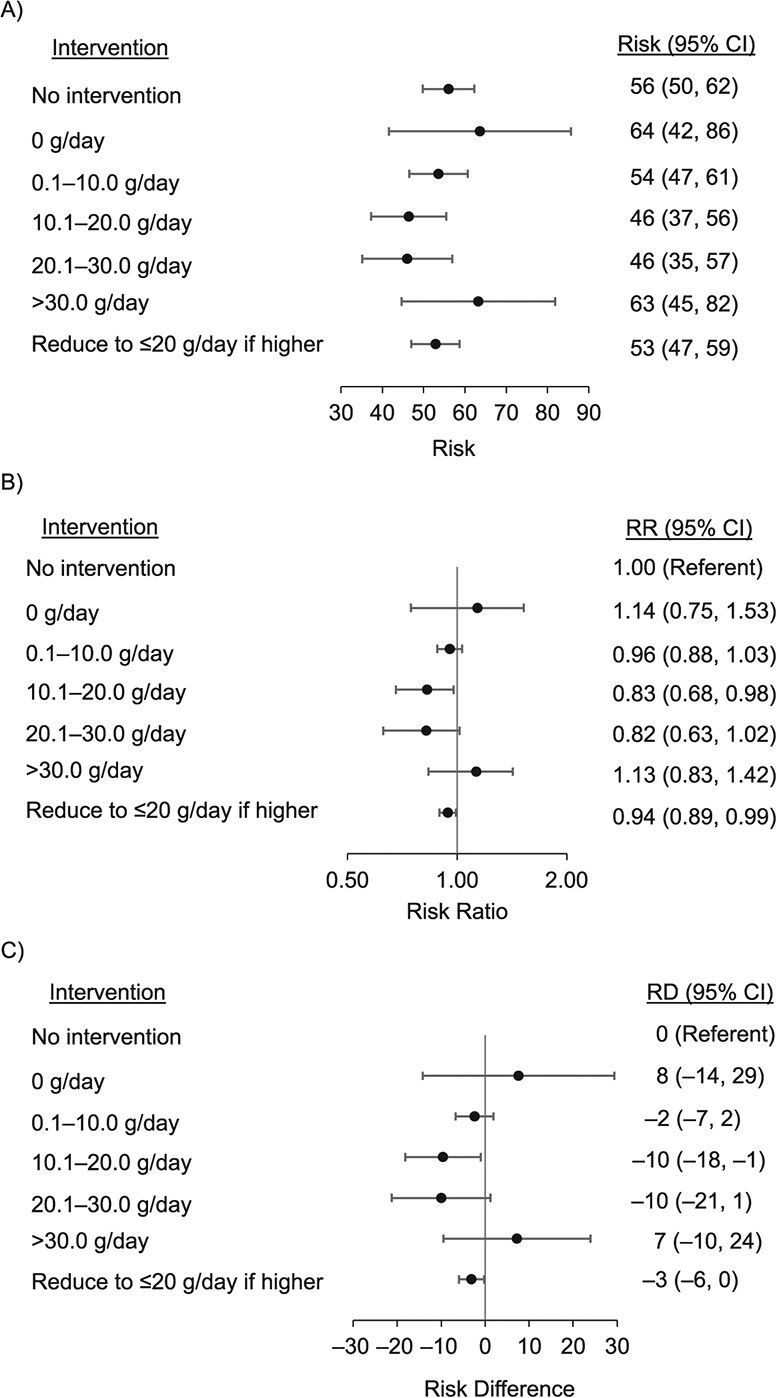
Estimated all-cause mortality over 20 years had the participants followed the hypothetical alcohol interventions (observational analogue to per-protocol analysis of the target trial), using data from the Australian Longitudinal Study on Women’s Health 1946–1951 Birth Cohort, 1996–2016. Risk and risk differences (RDs) are presented per 1,000 women, adjusted for the following time-fixed confounders: baseline age, country of birth, highest educational qualification, prebaseline smoking (smoked at least 100 cigarettes in total before baseline), alcohol intake (in late teens, twenties, and thirties), ever diagnosed with selected conditions (depression or anxiety, heart disease, hypertension, or diabetes) before baseline, and the following time-varying confounders: living in most socioeconomically disadvantaged areas, marital status, physical activity level, daily number of cigarettes, daily vegetable intake, body mass index, diagnoses or treatment of depression or anxiety since last survey, diagnoses or treatment of comorbidities since last survey (cancer, heart diseases, hypertension, or diabetes), and participants’ subjective general health. Average % intervened on was averaged over all waves. Observed risk was 56 per 1,000 women. The validity of estimates from the parametric g-formula relied on correct model specifications. The fact that we were able to closely reproduce the observed risks of death under the “no intervention” scenario (a necessary condition for no overall model misspecification) provided reassurance that our model specification was adequate overall. Average percentage intervened on are as follows: no intervention (0%), 0 g/day (83%), 0.1 to 10.0 g/day (50%), 10.1 to 20.0 g/day (77%), 20.1 to 30.0 g/day (90%), >30.0 g/day (92%), and reduce to ≤20 g/day if higher (14%). CI, confidence interval; RR, risk ratio.

**Figure 4:**
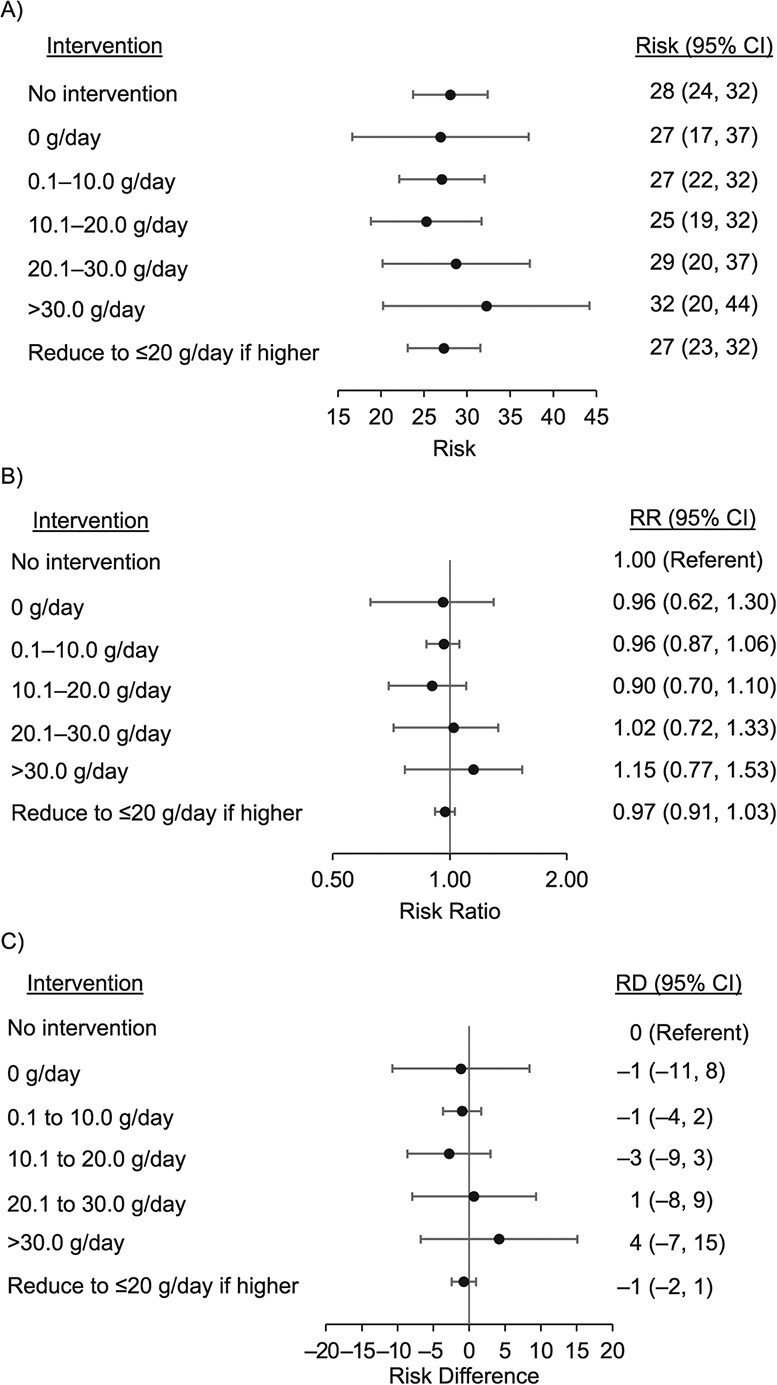
Estimated cancer mortality over 18 years had the participants followed the hypothetical alcohol interventions (observational analogue to per-protocol analysis of the target trial), using data from the Australian Longitudinal Study on Women’s Health 1946–1951 Birth Cohort, 1996–2016. Risk and risk differences (RDs) are presented for per 1,000 women, adjusted for the following time-fixed confounders: Baseline age, country of birth, highest educational qualification, prebaseline smoking (smoked at least 100 cigarettes in total before baseline), alcohol intake in late teens, twenties and thirties, ever diagnosed with selected conditions (depression or anxiety, heart disease, hypertension, or diabetes) before baseline, and the following time-varying confounders: living in most socioeconomically disadvantaged areas, marital status, physical activity level, daily number of cigarettes, daily vegetable intake, body mass index, diagnoses or treatment of depression or anxiety since last survey, diagnoses or treatment of comorbidities since last survey (cancer, heart diseases, hypertension, or diabetes), and participants’ subjective general health. Average % intervened on was averaged over all waves. Observed risk was 29 per 1,000 women. The validity of estimates from the parametric g-formula relied on correct model specifications. The fact that we were able to closely reproduce the observed risks of death under the “no intervention” scenario (a necessary condition for no overall model misspecification) provided reassurance that our model specification was overall adequate. Average percentage intervened on are as follows: no intervention (0%), 0 g/day (83%), 0.1 to 10.0 g/day (50%), 10.1 to 20.0 g/day (77%), 20.1 to 30.0 g/day (90%), >30.0 g/day (92%), and reduce to ≤20 g/day if higher (15%). CI, confidence interval; RR, risk ratio.

Figure 3 shows the 20-year all-cause mortality, and Figure 4 shows the 18-year cancer mortality, had the women sustainedly followed the assigned interventions. The estimated risks under no intervention were 56 and 28 per 1,000 women for all-cause and cancer mortality, respectively, which closely reproduced the observed risks of 56 and 29 per 1,000 women.

As shown in Figure 3, all-cause mortality was lowest under interventions of “10.1–20.0 g/day” and “20.1–30.0 g/day.” We observed 17%–18% lower all-cause mortality under interventions “10.1–20.0 g/day” (RR = 0.83, 95% CI: 0.68, 0.98; RD = –10, −18 to −1 per 1,000 women) and “20.1–30.0 g/day” (RR = 0.82, 95% CI: 0.63, 1.02; RD = –10, −21 to 1 per 1,000 women) compared with no intervention. The “0.1–10.0 g/day” intervention showed no difference in risk relative to no intervention. The estimated all-cause mortality under the sustained “0 g/day” and the “>30.0 g/day” interventions was higher than under no intervention, with wide CIs (for 0 g/day, RR = 1.14, 95% CI: 0.75, 1.53; RD = 8, −14 to 29 per 1,000 women; for >30.0 g/day, RR = 1.13, 95% CI: 0.83, 1.42; RD = 7, −10 to 24 per 1,000 women). Reducing drinking to ≤20 g/day, if higher, slightly lowered all-cause mortality compared with no intervention (RR = 0.94, 0.89, 0.99; RD = –3, −6 to 0 per 1,000 women).

The point estimate for cancer mortality was slightly lower under the “10.1–20.0 g/day” intervention (RR = 0.90, 0.70, 1.10; RD = –3, −9 to 3 per 1,000 women) and higher in the “>30.0 g/day” intervention (RR = 1.15, 0.77, 1.53; RD = 4, −7 to15 per 1,000 women) relative to no intervention, both with CIs crossing the null (Figure 4). Other interventions had marginally lower RRs and RDs that were imprecisely estimated. The estimates in Figures 3 and4 were robust to different modeling orders of covariates (Web Table 3).

**Table 5:** All-Cause and Cancer Mortality in Relation to Baseline Alcohol Intake, Regardless of Whether Individuals Continued on the Same Intake Level After Baseline (Baseline Analysis of the Target Trial), Using Data From the Australian Longitudinal Study on Women’s Health 1946–1951 Birth Cohort, 1996–2016

	**All-Cause Mortality**				**Cancer Mortality**			
**Baseline Daily Alcohol Intake, g/day**	**Person-Years**	**No. of Deaths** ^**a**^	**HR** ^**b**^	**95% CI**	**Person-Years**	**No. of Deaths** ^**a**^	**HR** ^**b**^	**95% CI**
0	23,710	86	1.00	Referent	21,499	45	1.00	Referent
0.1–10.0	97,775	286	0.88	0.65, 1.18	88,599	147	0.82	0.55, 1.22
10.1–20.0	39,218	97	0.84	0.59, 1.21	35,525	52	0.81	0.49, 1.32
20.1–30.0	16,236	44	0.85	0.56, 1.31	14,711	23	0.81	0.45, 1.45
>30.0	10,536	53	1.45	0.94, 2.24	9,570	25	1.33	0.72, 2.45

Abbreviations: CI, confidence interval; HR, hazard ratio.

^a^ Number of deaths in each category was averaged across 30 imputations because some death cases had missing alcohol data, and imputed alcohol intake values differ slightly in each imputation. Number of deaths (averaged total, *n* = 566) did not equal the observed number of deaths (*n* = 569) due to rounding.

^b^ Age was the timescale, adjusted for country of birth, highest educational qualification, alcohol consumption levels (in late teens, twenties, and thirties), ever smoked 100 or more cigarettes before baseline, self-reported history of selected conditions (depression, heart disease, and diabetes), and the following covariates measured in 1996: living in most socioeconomically disadvantaged areas, marital status, physical activity level, daily number of cigarettes, body mass index, and poor or fair subjective general health. Daily vegetable intake was not available in 1996 and 1998.

## DISCUSSION

Using data from the ALSWH 1946–1951 birth cohort, we emulated a target trial to estimate the effect of sustained alcohol interventions initiated in midlife on 20-year all-cause mortality and 18-year cancer mortality in women. Our baseline analysis showed weak evidence of lower mortality intakes up to 30 g/day and higher mortality for intakes above 30 g/day relative to abstention. A similar relationship was observed for all-cause mortality in the per-protocol analysis, with slightly more pronounced lower mortality seen for intakes of 10.1–30.0 g/day relative to the no-intervention scenario. This inverse relationship with intakes up to 30 g/day was less evident for cancer mortality, while evidence was also weak for higher mortality with intakes above 30 g/day in the per-protocol analysis.

The strengths of our study include multiple, frequent measurements of alcohol intake and potential confounders. We emulated a target trial on sustained alcohol interventions and mortality for a situation where randomized trials are infeasible. Using the parametric g-formula, we also better adjusted for time-varying confounding when estimating the observational analogue to the per-protocol effect of sustained alcohol interventions specified in the target trial, which provided estimates that are easy to interpret and more relevant for guiding policy making. Although time-varying Cox regression is a commonly used approach to handle exposure and covariates that change during follow-up, it requires stratification over time-varying confounders (i.e., estimates stratum-specific hazard ratios and averages the information-weighted hazard ratios) (26). This approach assumes no feedback between time-varying exposures and time-varying confounders (27). When time-varying confounders are affected by prior alcohol intake, this stratification-based approach for confounding adjustment would adjust away some of the mortality effect of alcohol consumption mediated through confounders that were also intermediates on causal pathways (as illustrated in Figure 1). A recent study showed that emulating a target trial and using g-methods to estimate effects from observational data produced estimates that were closer to benchmark results from randomized controlled trials than those that used time-varying Cox regression (28). Our study also contributes to the scarce literature on long-term alcohol consumption and mortality in women (29, 30).

Several limitations need to be considered. Although the parametric g-formula allows estimation of per-protocol effects without introducing biases of conditioning on a common effect or a mediator as a conventional regression analysis would, it does not eliminate other biases. We cannot exclude the possibility of unmeasured or residual confounding despite careful adjustment of available information on confounders to approximate randomization. For example, we had limited information on smoking history prior to baseline. Although we adjusted for whether the participants had smoked at least 100 cigarettes in their lives, more information on prebaseline smoking such as duration and intensity would be necessary to account for confounding from prior smoking. In addition, measurement error of exposure is likely because daily alcohol intake could only be approximated without information on beverage type. The baseline questionnaire also did not distinguish between lifetime abstainers and former drinkers. Therefore, we were unable to disentangle a mortality effect for these 2 groups. Although we adjusted for early-life alcohol consumption, the intakes were recalled in broad categories (Web Table 1) and therefore likely insufficient to control for earlier-life intake. Moreover, in the per-protocol analysis, residual confounding is possible due to participants’ health deterioration (31), which may not be fully captured by self-reported health conditions, even if these were assessed every 2–3 years. However, a simulation study showed that estimates were less biased using g-methods than when completely ignoring time-varying confounding (32).

A meta-analysis of observational studies on alcohol consumption over adulthood and mortality found a J-shaped relationship between alcohol intake over time and all-cause mortality in men, and found studies including women were scarce (29). A study of middle-aged adults in Melbourne (33) found a consistent J-shaped relationship between alcohol intake over mid-adulthood and all-cause mortality in women (lower mortality at <10 g/day of lifetime average intake; elevated mortality at higher intake levels relative to zero intake). Similarly, in the European Prospective Investigation into Cancer and Nutrition (EPIC) (34), female never-drinkers and those who drank ≥30 g/day (lifetime average) had higher mortality than those who drank 0.1–4.9 g/day; the HRs were 1.26 (95% CI: 1.18, 1.35) for never-drinkers and 1.27 (95% CI: 1.13, 1.43) for ≥30 g/day. Drinking ≥30 g/day was also associated with a higher mortality due to alcohol-related cancers (cancers of the upper aerodigestive tract, liver, colorectum, and female breast) in women (HR = 1.49, 95% CI: 1.07, 2.06). Findings from a study of older adults (69% women) in the United States (Prostate, Lung, Colorectal, and Ovarian (PLCO) Cancer Screening Trial) reported a consistent J-shaped association between lifetime alcohol consumption and mortality, with intakes below 1 drink per day being associated with the lowest total mortality (35). However, these studies do not provide information on intake patterns over time (e.g., increased, decreased, or sustained).

Some studies of women have assessed mortality outcomes of changes in alcohol consumption. A study of adults in California found that women who quit drinking had higher all-cause mortality than women who reported drinking in questionnaires 10 years apart (HR = 1.72, 95% CI: 1.11, 2.66) (36). A study of Swedish women reported that, compared with consistently low intake (<5 g/day), abstinence (HR = 1.53, 1.20, 1.96) and quitting (HR = 1.49, 95% CI: 1.10, 2.02) were associated with higher mortality, while maintaining ≥5 g/day of intake was not (30). However, the California study did not adjust for time-varying confounders, while the Swedish study adjusted for confounders measured at a follow-up visit, which could also be mediators of any effect of alcohol on mortality.

In cohort studies of lifetime average alcohol intake or intake changes over time, follow-up often began after exposure started. For example, in the PLCO Cancer Screening Trial study (35), follow-up started at the time when lifetime alcohol intake from age 18 to 55 years, or older, was recalled instead of the time when the exposure of interest started. This left-truncation of follow-up would bias the estimate because such analyses were restricted to people who survived until the truncation date (18). Using the target trial approach, we aligned the start of follow-up with start of the midlife alcohol interventions.

Our per-protocol analysis showed limited improvement in all-cause and cancer mortality in this cohort of women under an intervention of reducing alcohol consumption to no more than 2 standard drinks if the observed consumption was higher. The Australian National Health Surveys showed that only about 10% of women aged 45–64 consumed more than 2 standard drinks daily in 2017/2018, which was lower than the 14%–15% in our sample (37). Therefore, the effect of such intervention may be even smaller for women in this age range in the general population.

Our baseline analysis compared all-cause and cancer mortality in alcohol intake categories corresponding to the interventions specified in our target trial. It estimated mortality in relation to baseline alcohol intake, regardless of whether individuals continued on the same intake level during follow-up. On the other hand, our per-protocol analysis estimated mortality under sustained adherence to our specified alcohol interventions. Although the results from the two approaches are not directly comparable (baseline analysis estimated hazard ratios in comparison to abstention, whereas per-protocol analysis estimated risk differences and ratios in comparison to a “no intervention” scenario), we observed a less prominent “J-shaped” relationship in the per-protocol analysis. This attenuated relationship does not necessarily imply that associations previously found in other observational studies were overestimated; the sources of and strengths of bias are likely different for each study (e.g., selection bias, under- or overadjustment of confounding, left truncation of time zero, as previously discussed).

In summary, our findings suggest that all-cause mortality could have been slightly lower than observed if this cohort of women had some alcohol consumption (no more than 30 g/day) rather than no consumption, but the results on cancer mortality of sustained interventions to reduce alcohol intake were less pronounced. All-cause and cancer mortality over the long term might have been higher if these women consumed more than 3 standard drinks per day, but a lack of causal association is plausible given the imprecise estimates. The low volume of heavy drinkers in the cohort did not allow for a meaningful assessment of the impact of heavy drinking on mortality. Considering the limitations of our study, results should be interpreted with caution and reproduced by other studies. The target trial framework and contemporary statistical techniques can help address some biases, but it is unlikely that a single study design or analytical method can provide a definitive answer to a causal question. Answering important causal questions, such as whether low to moderate alcohol consumption lowers mortality, requires triangulating evidence from different causal study designs that are subject to different and unrelated forms of bias (38).

## Supplementary Material

Web_Material_kwad164
