# Supplementary material for: Sustained Hypothetical Interventions on Midlife Alcohol Consumption in Relation to All-Cause and Cancer Mortality: The Australian Longitudinal Study on Women’s Health
Source: Am J Epidemiol. 2023 Jul 24;193(1):75–86. doi: 10.1093/aje/kwad164 (PMC10773481; doi:10.1093/aje/kwad164)
Supplement: Web_Material_kwad164 [file web_material_kwad164.pdf]

Web Material

Sustained hypothetical interventions on midlife alcohol consumption in relation to all-cause and cancer mortality: The Australian Longitudinal Study on Women's Health

Yi Yang, Allison M. Hodge, Brigid M. Lynch, Pierre-Antoine Dugué, Elizabeth J. Williamson, Harindra Jayasekara, Gita Mishra, and Dallas R. English

Table of Contents

Web Figure 1. Timeline of the data collection waves for ALSWH 1946-51 birth cohort. 2

Web Table 1. Details of covariates and models in the parametric g-formula...................................3

Web Table 2. Baseline characteristics of eligible women, by death status 5

Web Table 3. Results from different orders of covariates under each scenario 7

Web Table 4. Number of all-cause and cancer deaths at each data collection wave.........................8

Web Appendix 1 9

Web Figure 1. Timeline of the data collection waves for ALSWH 1946-51 birth cohort.

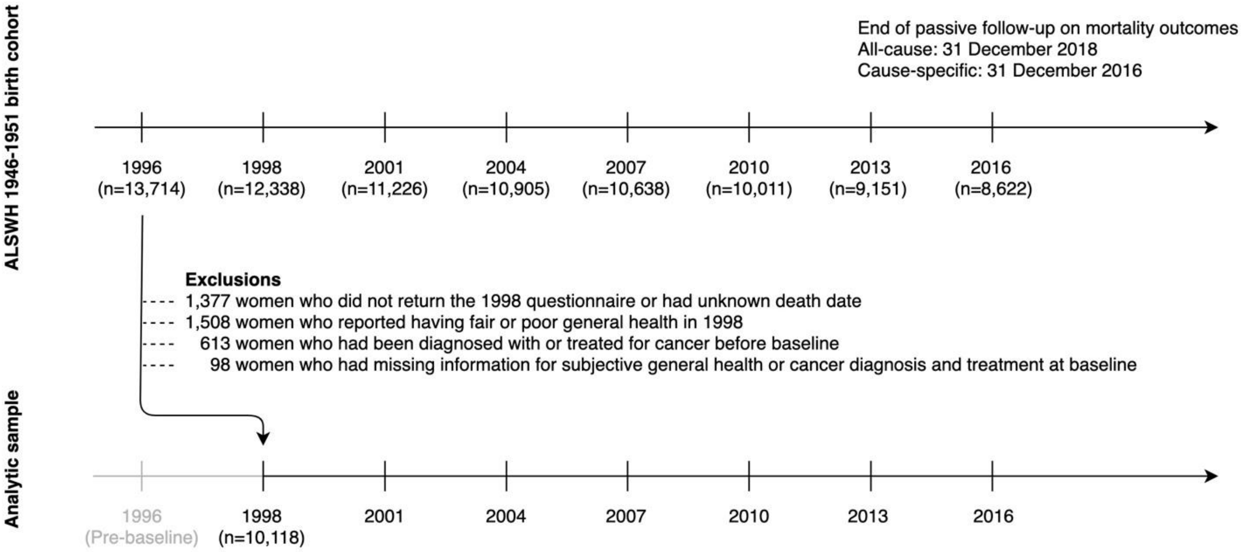

We used the 1998 wave as baseline to be able to adjust for pre-baseline confounders. In the analytic sample, multiple imputation by chained equation was used to impute missing data due to non-respondence of follow-up questionnaires or missing responses from respondents.

Web Table 1. Details of covariates and models in the parametric g-formula

Variables	Categories	Model when used as dependent variable	Variables conditioned on in the model	Functional form when used as independent variable
Alcohol intake	Continuous in g/day	Logistic model for indicator for any alcohol consumption (>0 g/day), and linear regression model for natural log of intake restricting to records where intake was greater than 0 g/day.	All time-fixed confounders, pre-baseline, and most recent lag value of time-varying confounders, and exposure history	Most recent lag value and cumulative average before it. For example, at time = k: value at k-1, and the average of g/day from time =0 to time=k-2
Death	Indicator	Logistic	As above.	-
Time-fixed confounders				
Age at baseline	Continuous in years	Not predicted	-	Continuous
Australian Born, as proxy for cultural exposure	Indicator	Not predicted	-	Indicator
Alcohol at different stage of life: average drink/week in your late teens, 20s, 30s (3 separate variables)	No alcohol; 1-7 drinks; 8-14 drinks; 15+ drinks	Not predicted	-	4 categories
Highest educational qualification	No formal education; higher school certificate; diploma/ university/higher degrees	Not predicted	-	3 categories
Smoked at least 100 cigarettes in total before baseline	Indicator	Not predicted	-	Indicator
Ever diagnosed with depression, heart disease, hypertension, or diabetes before baseline (one variable for each condition)	Indicator	Not predicted	-	Indicator
Time-varying confounders				
Area-based Index of Relative Socio-Economic Disadvantage (IRSD)	Indicator of the most disadvantaged quartile	Logistic	All time-fixed confounders, pre-baseline, and most recent lag of time-varying confounders, and exposure history	Binary indicator

**Variables**	**Categories**	**Model when used as dependent variable**	**Variables conditioned on in the model**	**Functional form when used as independent variable**
Married/de facto	Indicator	Logistic	As above.	Binary indicator
Physical activity	Continuous in weekly metabolic equivalent minutes (METmin/week)	Logistic model for indicator that METmin/week was greater than 0, and linear regression model for natural log of METmins/week restricting to records greater than 0.	As above.	Quadratic function
Number of cigarettes per day	Continuous in counts	Logistic model for indicator that number was greater than 0, and linear regression for natural log of number restricting to records greater than 0.	As above.	Quadratic function
Vegetable intake (serves/day), not available in 1996 and 1998 data collection.	<=1; 2 to 3; 4; ≥5	Ordered logistic	As above.	4 categories
Body mass index	Continuous in kg/m^2	Truncated normal regression with lower bound set to the minimum and maximum values of BMI in the observed dataset plus offset terms (+/- 0.01%).	As above.	Quadratic function
Diagnosed with or treated for depression or anxiety since last survey	Indicator	Logistic	As above.	Indicator
Diagnosed with or treated for any of the following conditions since last survey: heart diseases, hypertension, diabetes, cancer.	Indicator	Logistic	As above.	Indicator
Subjective general health	Indicator of fair or poor health	Logistic	As above.	Indicator

Web Table 2. Baseline characteristics of eligible women, by death status

**Characteristics**	**Overall (n=10118)**	**Alive (n=9549)**	**Dead (n=569)**
Alcohol intake categories, N(%)			
0 g/day	1176(12.7)	1100(12.6)	76(15.2)
>0 to 10 g/day	4815(52.1)	4567(52.2)	248(49.6)
>10 to 20 g/day	1926(20.8)	1841(21.1)	85(17.0)
>20 to 30 g/day	799(8.6)	759(8.7)	40(8.0)
>30 g/day	528(5.7)	477(5.5)	51(10.2)
Age, years, mean(SD)	49.5(1.5)	49.5(1.5)	49.7(1.4)
Born in Australia, N (%)	7688(76.7)	7249(76.7)	439(77.8)
Married or de facto, N(%)	8443(83.9)	7992(84.2)	451(80.0)
Highest qualification, N (%)			
No formal qualification	1570(15.6)	1449(15.3)	121(21.5)
School certificate	4951(49.3)	4678(49.4)	273(48.5)
Diploma/University/Higher degree	3516(35.0)	3347(35.3)	169(30.0)
Area-based Index of Relative Socio-Economic Disadvantage, N(%)			
Most disadvantaged quartile	2391(23.6)	2260(23.7)	131(23.0)
Smoking status, N(%)			
Never	5481(57.6)	5254(58.4)	227(43.2)
Former	2546(26.7)	2424(26.9)	122(23.2)
Current	1495(15.7)	1319(14.7)	176(33.5)
Cigarettes per day for current smoker, n/day, median (IQR)	20.0(12.0,25.0)	20.0(12.0,25.0)	20.0(15.0,30.0)
Physical activity, N(%)			
Very low (0 to <33.3 METmin/week)	1471(15.8)	1372(15.6)	99(19.7)
Low (33.3 to <500 METmin/week)	2626(28.3)	2487(28.3)	139(27.7)
Moderate (500 to <1000 METmin/week)	2219(23.9)	2113(24.1)	106(21.1)
High (>=1000 METmin/week)	2965(31.9)	2807(32.0)	158(31.5)
Body mass index, kg/m2, mean(SD)	26.1(5.1)	26.1(5.1)	26.7(5.7)
Subjective general health, N(%)			
Excellent	1485(14.7)	1429(15.0)	56(9.8)

Very good	4375(43.2)	4146(43.4)	229(40.2)
Good	4258(42.1)	3974(41.6)	284(49.9)
Ever had the following conditions, N(%)			
Depression or anxiety	1023(10.1)	967(10.1)	56(9.8)
Heart diseases	55(0.5)	49(0.5)	6(1.1)
Hypertension	841(8.3)	782(8.2)	59(10.4)
Diabetes	82(0.8)	76(0.8)	6(1.1)

Abbreviations: N, number; SD, standard deviation; IQR, inter-quartile range; MET, metabolic equivalent.

Web Table 3. Results from different orders of covariates under each scenario

**Interventions**	All-cause Mortality Risk, per 1000 women (95%CI)			Cancer Mortality Risk, per 1000 women (95%CI)		
	Main model	Order A	Order B	Main model	Order A	Order B
No intervention	56.0(49.8,62.2)	55.8(49.7,61.9)	55.7(49.6,61.8)	28.1(23.7,32.4)	28.1(23.9,32.2)	27.8(23.7,31.9)
0g/day	63.6(41.6,85.7)	63.6(41.5,85.6)	63.5(41.4,85.5)	26.9(16.6,37.2)	26.9(16.9,36.9)	26.8(16.8,36.8)
>0 to 10 g/day	53.6(46.5,60.7)	53.3(46.3,60.4)	53.3(46.2,60.5)	27.1(22.1,32.0)	27.1(22.3,31.9)	27.0(22.3,31.8)
>10 to 20 g/day	46.4(37.3,55.5)	46.2(37.3,55.1)	46.1(37.2,55.1)	25.3(18.8,31.7)	25.2(18.9,31.5)	25.2(18.9,31.5)
>20 to 30g/day	46.0(35.1,57.0)	45.8(35.1,56.6)	45.8(35.1,56.6)	28.7(20.2,37.3)	28.7(20.4,37.1)	28.7(20.3,37.0)
>30 g/day	63.2(44.6,81.8)	63.0(44.8,81.3)	62.9(44.8,81.1)	32.2(20.2,44.2)	32.2(20.5,43.9)	32.1(20.5,43.7)
Reduce to ≤ 20 g/day if higher	52.9(47.0,58.7)	52.7(47.0,58.5)	52.6(46.8,58.3)	27.3(23.1,31.5)	27.4(23.4,31.4)	27.2(23.3,31.2)

Ordering of covariates: Main Model - Alcohol, socioeconomic disadvantage, marital status, lifestyle factors (physical activity, daily number of cigarettes, vegetable intake, body mass index), health (comorbidities and subjective health); Order A - Alcohol, socioeconomic disadvantage, marital status, health, lifestyle factors; Order B - Alcohol, health, lifestyle factors, socioeconomic disadvantage, marital status.

Web Table 4. Numbers of all-cause and cancer deaths at each data collection wave

Outcome	Number of deaths								End of follow-up
	1998	2001	2004	2007	2010	2013	2016	Post-2016	
All-cause deaths	0	17	66	125	212	322	444	444	31 December 2018
Cancer deaths	0	12	40	81	137	214	292	-	31 December 2016

Web Appendix 1

Alcohol data from the 2001 and 2013 waves

In 2001 and 2013, individuals completed a separate food frequency questionnaire (FFQ), which included questions about consumption of specific types of alcoholic beverages. The amount of each alcoholic beverage (beer, wine, spirits) was multiplied by the daily equivalent frequency and alcohol content to calculate the daily alcohol intake.[1] As the 2001 questionnaire did not include the frequency and quantity questions, we calibrated the daily alcohol intake calculated from the FFQ using regression calibration based on the 2013 data (where alcohol questions were asked in both the main questionnaire and the FFQ): for current drinkers in 2001, $\textit{alcohol intake} = \alpha + \lambda * \textit{FFQ intake}$, where α and λ were estimated from linear regression of the 2013 questionnaire-estimated intake against 2013 FFQ-estimated intake.[2]

Multiple imputation using chained equations

We included all variables to be used in the parametric g-formula and the Cox models, and auxiliary variables that were correlated (Pearson's $r>0.6$) with the variables that had missing data. After taking into account their responses at other waves and their fully observed mortality status, we assumed the data were missing at random, i.e., the probability of data being missing did not depend on the unobserved data, conditional on the observed data. We used 30 imputations for all analyses. In the parametric g-formula analyses, point estimates for each intervention were averaged over all imputed datasets; bootstrap samples were drawn for each imputed data set to estimate the standard errors; 95% CIs were calculated using Rubin's rules. The imputation of missing data relied on the missing-at-random assumption. We believed that the key drivers of missingness were likely captured in the data included in the imputation models.

References

1. Hodge A, English D, Itsiopoulos C, O'Dea K, Giles G. Does a Mediterranean diet reduce the mortality risk associated with diabetes: evidence from the Melbourne Collaborative Cohort Study. *Nutrition, Metabolism and Cardiovascular Diseases* 2011; **21**: 733-9.

2. Rosner B, Willett WC, Spiegelman D. Correction of logistic regression relative risk estimates and confidence intervals for systematic within-person measurement error. *Stat Med* 1989; **8**: 1051-69; discussion 71-3.
